# Hyperfine-Resolved Near-Infrared Spectra of H_2_^17^O

**DOI:** 10.1021/acs.jpca.1c05681

**Published:** 2021-09-02

**Authors:** Mattia Melosso, Meissa L. Diouf, Luca Bizzocchi, Michael E. Harding, Frank M. J. Cozijn, Cristina Puzzarini, Wim Ubachs

**Affiliations:** † Dipartimento di Chimica “Giacomo Ciamician”, Università di Bologna, Via Selmi 2, 40126 Bologna, Italy; ‡ Department of Physics and Astronomy, LaserLab, Vrije Universiteit, De Boelelaan 1081, 1081 HV Amsterdam, The Netherlands; § Scuola Normale Superiore, Piazza dei Cavalieri 7, 56126 Pisa, Italy; ∥ Institut für Nanotechnologie, Karlsruher Institut für Technologie (KIT), Campus Nord, Postfach 3640, 76021 Karlsruhe, Germany

## Abstract

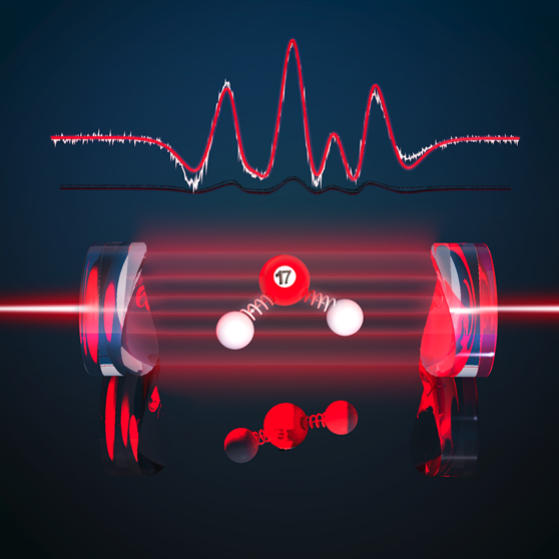

Huge efforts have recently been taken in the derivation of accurate compilations of rovibrational energies of water, one of the most important reference systems in spectroscopy. Such precision is desirable for all water isotopologues, although their investigation is challenged by hyperfine effects in their spectra. Frequency-comb locked noise-immune cavity-enhanced optical-heterodyne molecular spectroscopy (NICE-OHMS) allows for achieving high sensitivity, resolution, and accuracy. This technique has been employed to resolve the subtle hyperfine splittings of rovibrational transitions of H_2_^17^O in the near-infrared region. Simulation and interpretation of the H_2_^17^O saturation spectra have been supported by coupled-cluster calculations performed with large basis sets and accounting for high-level corrections. Experimental ^17^O hyperfine parameters are found in excellent agreement with the corresponding computed values. The need of including small hyperfine effects in the analysis of H_2_^17^O spectra has been demonstrated together with the ability of the computational strategy employed for providing quantitative predictions of the corresponding parameters.

## Introduction

Hyperfine parameters constitute an important source of information on physicochemical molecular properties related to electron densities and electronic structure. The nuclear quadrupole coupling is the strongest hyperfine interaction in closed-shell molecules. From the associated constants, information on intramolecular interactions and on the ionic or π character of the bonds involving the quadrupolar nucleus can be retrieved. Nuclear spin-rotation interaction and their corresponding constants provide, instead, insight into the paramagnetic part of the nuclear magnetic shielding constants, and this was the motivation for investigating the hyperfine structure (HFS) of the rotational spectrum of water isotopologues containing an ^17^O.^1,2^ Nuclear quadrupole coupling and spin-rotation interaction give rise to splittings in the rotational and rovibrational transitions (the HFS mentioned above), thus providing an experimental approach to the abovementioned molecular properties.

However, the hyperfine effects in water tend to be small in view of its closed-shell nature in the electronic ground state, thus requiring a very high resolving power to reveal subtle splittings in its spectrum. Indeed, the HFS of the H_2_^17^O rotational spectrum was resolved only in a few investigations. Besides the work reported in ref (1), there is only the study by De Lucia and Helminger from the 1970s.^3^ Both works concerned the vibrational ground state and were performed via pure rotational spectroscopy in the microwave domain. However, to the best of our knowledge, the HFS of the ^17^O-containing species has never been resolved in vibrational excited states. Water is a crucial reference spectroscopic system, and a comprehensive characterization is also essential for this isotopic species. As in the main isotopologue, the rovibrational states are separated into two subsets: the ortho and para nuclear-spin isomers. Ortho–para conversion represents an open issue, with the energy separation of the ortho and para states requiring to be known precisely. In the case of H_2_^17^O, a precise determination of the rovibrational level structure cannot avoid the full characterization of its HFS because the hyperfine splittings are of the same order of magnitude as the desired accuracy.

In the past decade, the development of intracavity-based optical techniques allowed high resolution and high sensitivity to be achieved also in vibrational spectroscopy. Different groups have applied such approaches to the investigation of vibrational excitation of H_2_^17^O.^4−7^ A large number of spectral lines were accurately measured in a wide spectral region, thereby leading to the revised and updated W2020 database of rovibrational transitions and energy levels of ^17^O-containing water.^8^ However, all of these studies were Doppler-limited and no hyperfine effects could be resolved. Recently, the intracavity techniques were further developed and combined with high accuracy calibration, thus allowing the exploitation of the Lamb-dip effect in rovibrational spectra.^9−12^ In only one study, such Lamb-dip vibrational spectroscopy was applied to H_2_^17^O, although the linewidth, due to collisional and time-of-flight broadening, was still too large to resolve the HFS.^13^

In the present study, we extend our previous work on the saturation spectroscopy of water^14^ to the ^17^O-containing species, thereby using the advanced capabilities of a frequency-comb locked noise-immune cavity-enhanced optical-heterodyne molecular spectroscopy (NICE-OHMS) setup to resolve, for the first time, the HFS of rovibrational transitions of H_2_^17^O. NICE-OHMS is a precision-spectroscopy technique that brings a new perspective in the field; having the sensitivity to observe saturation spectra at very low pressures and powers, it allows for the resolution of hyperfine structures in vibrational overtones, while at the same time providing an absolute frequency scale at kHz accuracy. The experimental determination of the hyperfine parameters for the vibrational state involved is supported and guided by state-of-the-art *ab initio* computations.

## Theory and Computations

Each rovibrational energy level of H_2_^17^O, including the HFS caused by the ^17^O nuclear spin, is uniquely labeled by seven quantum numbers. They can be classified as vibrational quantum numbers, (*v*_1_*v*_2_*v*_3_), representing the excitation of the symmetric stretching, bending, and antisymmetric stretching modes, respectively, and the rotational quantum numbers. These are the total angular momentum for the end-over-end rotation *J*, the pseudoquantum numbers *K*_a_ and *K*_c_ used in the designation of levels in asymmetric-top rotors, and *F* the total angular momentum accounting for the coupling of *J* with the ^17^O nuclear spin (*I* = 5/2). The latter coupling, which is caused by the interactions mentioned above, splits each rotational level into different but closely spaced sublevels. For *ortho*-H_2_^17^O, additional splittings are due to the hydrogen spins (only spin-rotation interaction), with the involved interaction being too small to produce measurable effects in the present experiment. The splittings of the energy levels generate the already mentioned HFS of the spectrum. While the HFS due to ^17^O exhibits several components typically separated by a few hundred kHz, that due to the hydrogen nuclei is expected only to give rise to a small broadening of the Lamb-dips, as already observed in a previous study on H_2_^16^O.^14^

To simulate the HFS of rovibrational transitions, the hyperfine parameters of each vibrational state involved in the transition are required. In particular, relying on the additivity approximation, vibrationally averaged parameters are evaluated as the sum of their equilibrium value and the corresponding vibrational correction, with the former term computed by means of a composite scheme. The hyperfine parameters under consideration are the oxygen (^17^O) quadrupole-coupling constants, χ_*ii*_, and the spin-rotation constants of both oxygen and hydrogen, *C*_*ij*_ (*i* and *j* denote the principal inertia axes).

The nuclear quadrupole coupling and nuclear spin-rotation constants have been computed adopting a similar protocol as in refs (1, 2). The so-called equilibrium values were evaluated at the semiexperimental equilibrium geometry^a^ (*r*(OH) = 0.9575 Å, ∠(HOH) = 104.51^°^)^15^ using the coupled-cluster singles and doubles approach augmented by a perturbative treatment of triple excitations (CCSD(T))^16−19^ in conjunction with the aug-cc-pCV6Z^20,21^ basis set. The CCSD(T) values have been augmented by higher-level corrections. These latter were evaluated as the difference between coupled-cluster singles, doubles, and triples (CCSDT)^22−24^ and CCSD(T) employing the aug-cc-pCV*X*Z (*X* = T, Q)^25−27^ basis sets (Δ*T* term), as well as the difference between coupled-cluster singles, doubles, triples, and quadruples (CCSDTQ)^22,23^ and CCSDT employing the aug-cc-pCV*X*Z (*X* = D, T)^25−27^ basis sets (Δ*Q* term). In the calculations of spin-rotation tensors, perturbation-dependent basis functions were used to ensure fast basis-set convergence, as described in refs (28, 29). Scalar relativistic corrections to the nuclear quadrupole-coupling tensors (ΔREL term) have been evaluated using second-order direct perturbation theory (DPT2)^30^ at the CCSD(T)/aug-cc-pCV6Z level.

As in previous studies focusing on the (000) vibrational ground state,^1,2^ the DVR-QAK^31^ scheme has been employed to compute the vibrational corrections to equilibrium values. In this approach, the treatment of vibrational effects is based on the variation principle and on the use of the so-called Watson Hamiltonian,^32^ given in terms of rectilinear dimensionless normal coordinates, and fully accounts for Coriolis interactions and anharmonic effects in the potential. Converged vibrationally averaged property values were determined by evaluating the corresponding expectation values over the vibrational wave function of the corresponding vibrational state, using a multidimensional Gauss–Hermite quadrature. For the actual computations, 11–15 quadrature points per each mode have been used to evaluate the matrix elements over the anharmonic part of the potential and 11–15 harmonic-oscillator basis functions per each mode have been used as a product basis. The vibrational corrections were calculated as the difference between the vibrationally averaged and the equilibrium values, both at the CCSD(T) level in conjunction with the aug-cc-pCVQZ^25−27^ basis set. A value of −25.58(22) mb was used for the ^17^O nuclear quadrupole moment.^33^

All computations were carried out with all electrons included in the correlation treatment and using the CFOUR program package;^34,35^ for some calculations, the parallel version of CFOUR(36) has been employed. All CCSDT and CCSDTQ results were obtained with the string-based many-body code MRCC(37,38) interfaced to CFOUR.

The equilibrium values and their vibrational correction terms are presented in Table 1 for the χ_*ii*_(^17^O)’s and in Tables 2 and 3 for the *C*_*ij*_’s of oxygen and hydrogen, respectively. These parameters have been computed for the (000) vibrational ground state as well as for the (040), (120), (021), (021), (200), (101), and (002) vibrational states. All these levels lie around 7000 cm^–1^, which is the frequency region explored in the present experiment.

**Table 1:** Equilibrium Values, Vibrational Corrections, and Vibrationally Corrected Values of the Oxygen Quadrupole-Coupling Tensor (NQCT) (MHz)

	*χ*_aa_	*χ*_bb_	*χ*_cc_
Equilibrium values^a^			
CCSD(T)^b^	–8.809	–1.066	+9.876
Δ*T*^c^	–0.012	–0.001	+0.012
Δ*Q*^d^	+0.011	–0.001	–0.010
ΔREL^e^	–0.009	–0.014	+0.023
sum^f^	–8.819	–1.082	+9.901
Vibrational corrections (DVR-QAK)^g^			
(000)	+0.007	–0.223	+0.216
(040)	–0.067	+0.177	–0.111
(120)	+0.023	–0.353	+0.329
(021)	+0.067	–0.415	+0.348
(200)	–0.005	–0.698	+0.704
(101)	+0.023	–0.735	+0.712
(002)	+0.040	–0.760	+0.719
Vibrationally corrected values^h^			
(000)	–8.812	–1.305	+10.117
(040)	–8.886	–0.905	+9.790
(120)	–8.796	–1.435	+10.230
(021)	–8.752	–1.497	+10.249
(200)	–8.824	–1.780	+10.605
(101)	–8.796	–1.817	+10.613
(002)	–8.779	–1.842	+10.620

a Computed at the semiexperimental geometry.

b Computed employing the aug-cc-pCV6Z basis set.

c Difference of the NQCT at CCSDT/aug-cc-pCVQZ and CCSD(T)/aug-cc-pCVQZ levels.

d Difference of the NQCT at CCSDTQ/aug-cc-pCVTZ and CCSDT/aug-cc-pCVTZ levels.

e Computed via DPT2 at the CCSD(T)/aug-cc-pCV6Z level.

f Sum of CCSD(T), Δ*T*, Δ*Q*, and ΔREL.

g Difference of vibrationally averaged and equilibrium value at the CCSD(T)/aug-cc-pCVQZ level.

h Equilibrium values augmented by the corresponding vibrational corrections.

**Table 2:** Equilibrium Values, Vibrational Corrections, and Vibrationally Corrected Values of the Oxygen Spin-Rotation Tensor (SRT) (kHz)

	*C*_aa_	*C*_bb_	*C*_cc_
Equilibrium values^a^			
CCSD(T)^b^	–22.25	–25.20	–17.48
Δ*T*^c^	–0.10	–0.11	–0.06
Δ*Q*^d^	+0.05	+0.08	+0.03
sum^e^	–22.31	–25.22	–17.50
Vibrational corrections (DVR-QAK)^f^			
(000)	–6.35	–2.78	–1.02
(040)	–25.86	–2.38	+3.13
(120)	–22.93	–5.53	–0.10
(021)	–20.05	–6.82	–1.24
(200)	–18.58	–7.79	–3.59
(101)	–16.80	–8.73	–4.40
(002)	–14.45	–9.67	–4.96
Vibrationally corrected values^g^			
(000)	–28.66	–28.01	–18.52
(040)	–48.16	–27.61	–14.37
(120)	–45.23	–30.75	–17.60
(021)	–42.35	–32.04	–18.74
(200)	–40.89	–33.01	–21.09
(101)	–39.10	–33.96	–21.91
(002)	–36.75	–34.89	–22.46

a Computed at the semiexperimental geometry.

b Computed employing the aug-cc-pCV6Z basis set.

c Difference of the SRT at CCSDT/aug-cc-pCVTZ and CCSD(T)/aug-cc-pCVTZ levels.

d Difference of the SRT at CCSDTQ/aug-cc-pCVDZ and CCSDT/aug-cc-pCVDZ levels.

e Sum of CCSD(T), Δ*T*, and Δ*Q*.

f Difference of vibrationally averaged and equilibrium value at the CCSD(T)/aug-cc-pCVQZ level.

g Equilibrium values augmented by the corresponding vibrational corrections.

**Table 3:** Equilibrium Values, Vibrational Corrections, and Vibrationally Corrected Values of the Hydrogen Spin-Rotation Tensor (SRT) (kHz)

	*C*_aa_	*C*_bb_	*C*_cc_	*C*_ab_^a^	*C*_ba_^a^
Equilibrium values^b^					
CCSD(T)^c^	–35.45	–31.78	–33.98	–47.81	–21.01
Δ*T*^d^	+0.00	+0.00	–0.01	–0.03	–0.02
Δ*Q*^e^	–0.01	+0.00	–0.01	–0.03	+0.00
sum^f^	–35.47	–31.78	–33.99	–47.87	–21.03
Vibrational corrections (DVR-QAK)^g^					
(000)	+1.24	+0.62	+1.51	–1.29	–0.09
(040)	+6.22	+0.43	+3.83	–26.56	+3.13
(120)	+4.24	+0.94	+3.08	–9.84	+1.30
(021)	+4.87	+1.65	+4.54	–9.07	+0.61
(200)	+2.69	+1.53	+2.65	–0.24	–0.32
(101)	+3.01	+2.08	+3.85	–0.05	–0.93
(002)	+3.10	+2.60	+4.88	+0.22	–1.51
Vibrationally corrected values^h^					
(000)	–34.23	–31.16	–32.48	–46.16	–21.13
(040)	–29.24	–31.36	–30.16	–74.44	–17.91
(120)	–31.23	–30.85	–30.91	–57.71	–19.73
(021)	–30.60	–30.13	–29.45	–56.94	–20.43
(200)	–32.78	–30.25	–31.34	–48.11	–21.35
(101)	–32.46	–29.70	–30.14	–47.93	–21.97
(002)	–32.37	–29.19	–29.11	–47.66	–22.55

a Please note that for the other hydrogen, the *C*_ab_ and *C*_ba_ terms are inverted in sign.

b Computed at the semiexperimental geometry.

c Computed employing the aug-cc-pCV6Z basis set.

d Difference of the SRT at CCSDT/aug-cc-pCVTZ and CCSD(T)/aug-cc-pCVTZ levels.

e Difference of the SRT at CCSDTQ/aug-cc-pCVDZ and CCSDT/aug-cc-pCVDZ levels.

f Sum of CCSD(T), Δ*T*, and Δ*Q*.

g Difference of vibrationally averaged and equilibrium value at the CCSD(T)/aug-cc-pCVQZ level.

h Equilibrium values augmented by the corresponding vibrational corrections.

## Experiment

**Figure 1:**
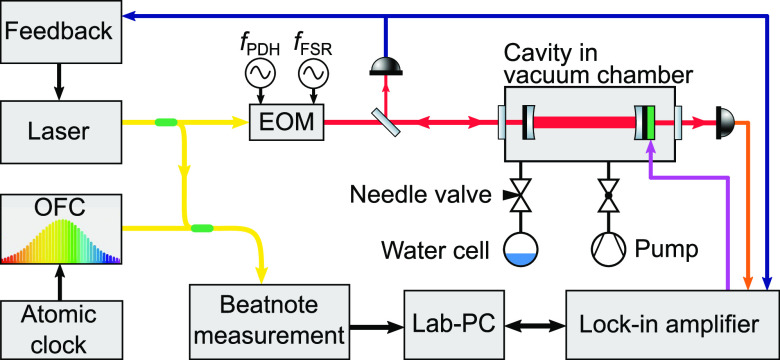
Layout of the experimental setup (for details see the text).

The measurements were performed in the near-infrared region employing the NICE-OHMS apparatus developed for saturation spectroscopy and already employed for HD^39^ and the main water isotopologue.^14^ In the present setup, shown schematically in Figure 1, a high-finesse (∼150 000) cavity has been employed together with an infrared diode laser operating at 1.4 μm. This laser is modulated at 305 MHz, equivalent to the free spectral range (FSR) of the cavity, for generating the side-band signals, and at 20 MHz for the cavity-lock via a Pound–Drever–Hall (PDH) stabilization scheme. In addition to the first layer of modulation at 305 MHz, the second layer of modulation is applied by dithering one of the cavity mirrors at a low frequency of 415 Hz. The doubly modulated spectroscopic signal is demodulated by a powerful lock-in system (Zurich Instruments; HF2LI), where the 1st and 3rd harmonics (*1f* and *3f* signals, respectively) of the dither modulation are extracted. A stabilized optical frequency-comb (OFC), referenced to a Cs atomic clock, is employed to stabilize the infrared laser and the optical cavity, and to provide a frequency scale accurate to the 1 kHz level.

The intracavity high-power laser enabled the saturation of several rovibrational transitions of H_2_^17^O and thus the exploitation of the Lamb-dip effect with extremely narrow dip profiles. Indeed, thanks to the highly reflective mirrors, the intracavity power can be increased up to 150 W. However, the intracavity power is matched to the oscillator strength to operate in the weakly saturating regime, thus avoiding significant power broadening. This results in intracavity powers below 1 W. The resulting linewidth of the resolved hyperfine components is mainly limited by the transit time of the molecules across the laser beam, overall yielding a width of ∼400 kHz (full width at half-maximum).

For the spectral recordings, an enriched H_2_^17^O sample (Cambridge Isotopes; 20% H_2_^17^O isotopic purity) has been used. The measurements have been carried out under steady gas flow conditions, at pressures in the range of 0.1–0.3 Pa, measured by a capacitance pressure gauge.

**Figure 2:**
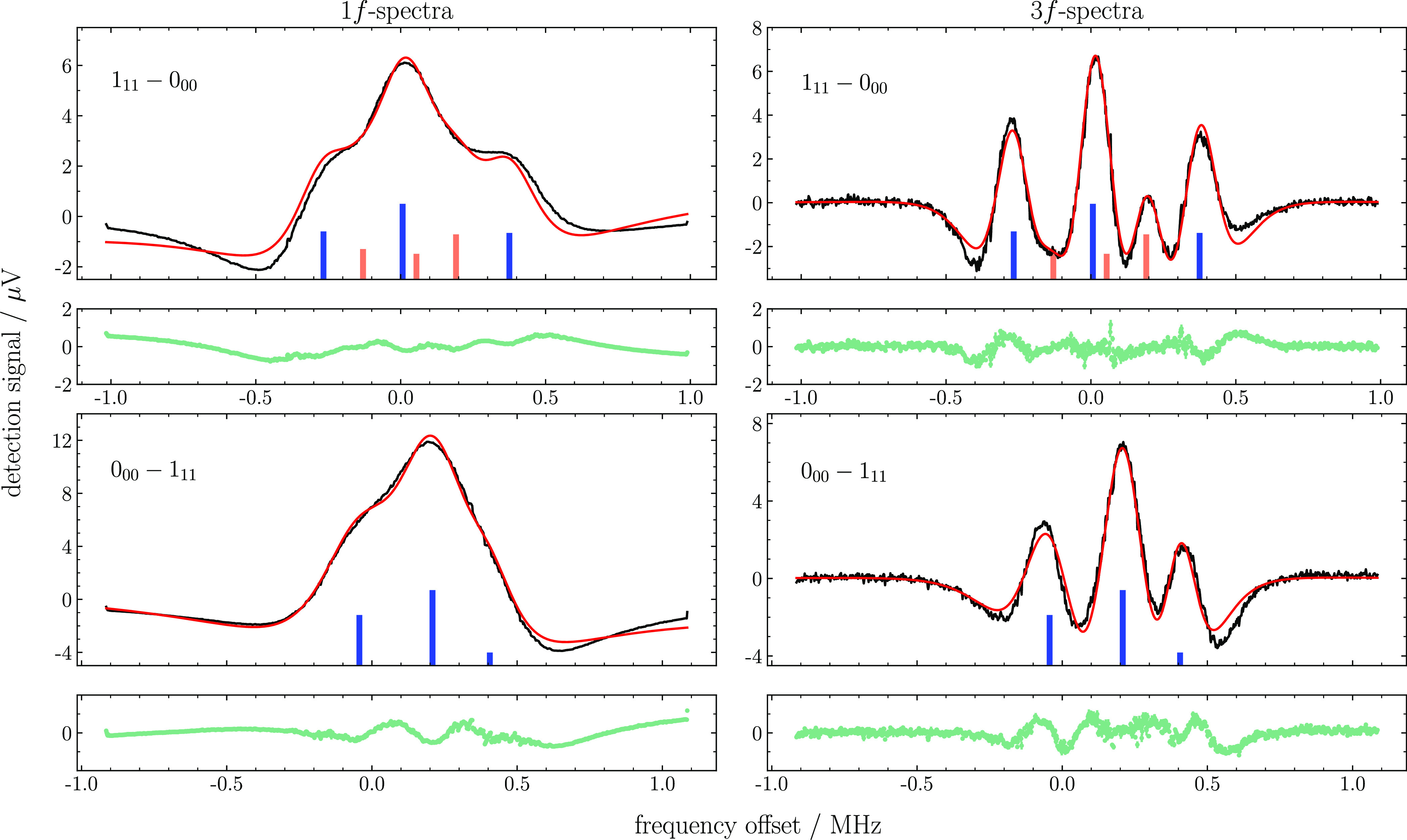
Modeling results for the *1f* and *3f* spectra recorded for the *JKaKc* = 1_11_ ← 0_00_ (top panels) and the *JKaKc* = 0_00_ ← 1_11_ (bottom panels) transitions of the (200) ← (000) overtone. The black and red lines plot the experimental data and the “best-fit” model, respectively. The stick spectra denote the positions and relative intensities of the hyperfine components (in blue) and crossover resonances (orange). The fit residuals (light green) are shown in the boxes below each panel.

**Figure 3:**
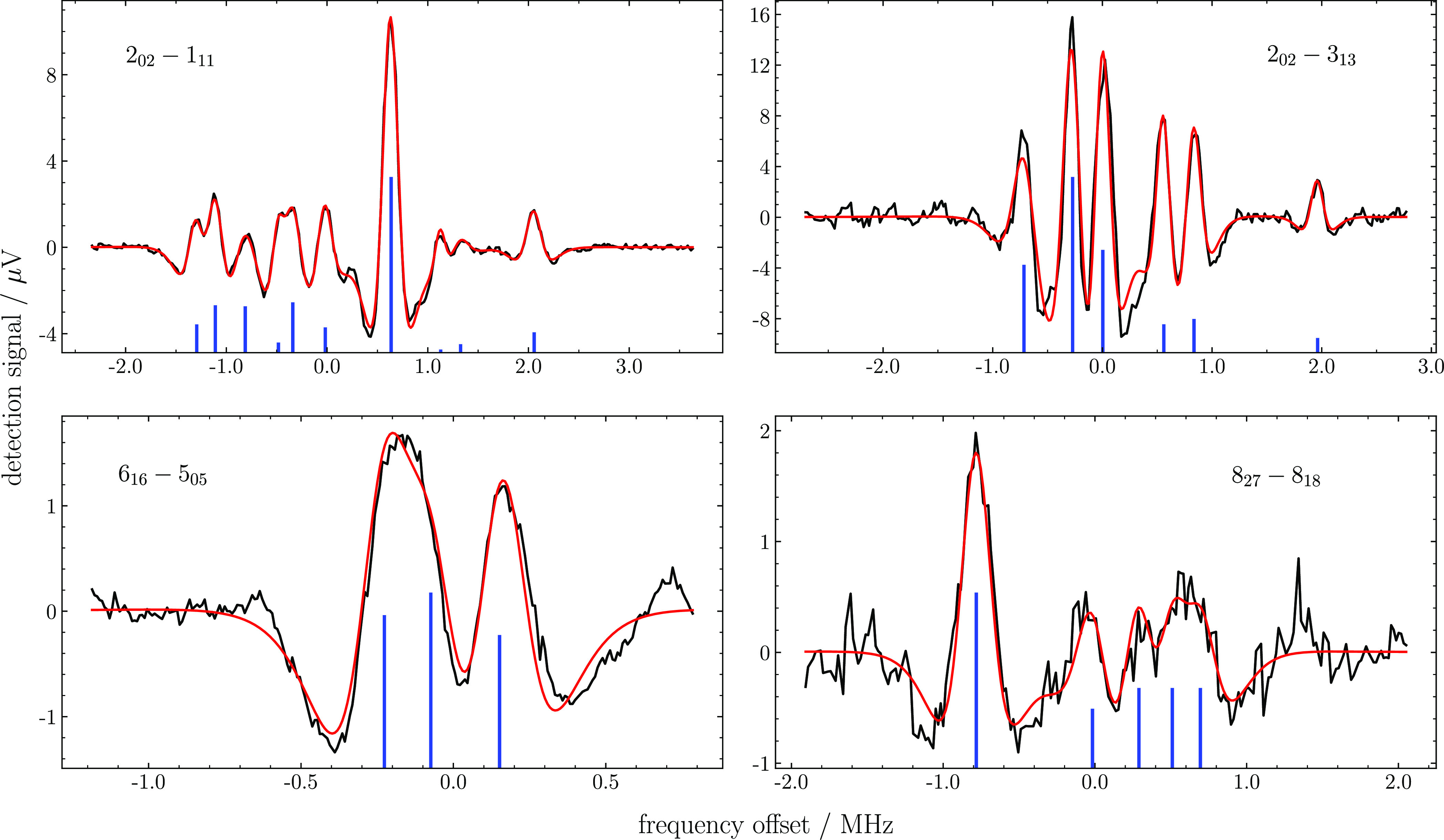
Experimental recordings and modeling results for the *3f* spectra of the *JKaKc* = 2_02_ ← 1_11_ (top-left), *JKaKc* = 2_02_ ← 3_13_ (top-right), *JKaKc* = 6_16_ ← 5_05_ (bottom-left), and *JKaKc* = 8_27_ ← 8_18_ (bottom-right) transitions of the (200) ← (000) overtone. The two top spectra pertain to the para-species, while the two bottom spectra to the ortho-species. The black and red lines plot the experimental data and the “best-fit” model, respectively. The stick spectra (blue bars) show the positions and relative intensities of the hyperfine components.

Among the vibrational states investigated computationally, the (200) state has been chosen in view of the intensity of its transitions. A number of saturation spectra were recorded for the (200) ← (000) rovibrational band at 7200 cm^–1^. In Figure 2, two spectra are shown: the *JKaKc* = 1_11_ ← 0_00_*R*-transition from the ground state para-level and the *JKaKc* = 0_00_ ← 1_11_ P-transition probing the lowest para-level in the (200) vibrationally excited state. Both *1f* and *3f* recordings are depicted, thereby demonstrating the superior resolution obtainable with the 3f demodulation scheme. Figure 3 shows four additional Lamb-dip spectra recorded in the high-resolution 3f mode and involving transitions for both *para*- and *ortho*-H_2_^17^O.

## Results and Discussion

Initially, we simulated the spectra using the experimental hyperfine constants previously determined for the ground state,^1^ and the *ab initio* values computed in this work for the (200) upper state. Simulations of the Lamb-dip spectra based on these “first guess” values were already able to well reproduce the experimental recordings. Nevertheless, as found in the Lamb-dip investigation of the ground-state rotational spectrum,^1^ additional features due to crossover resonances were occasionally observed. These are also referred to as “ghost transitions” and are a well-known effect in Lamb-dip saturation spectra.^40^ They appear in the case of transitions with a common state (either upper or lower) and partially overlapping Doppler profiles. Here, we adopt a phenomenological treatment of such features, whose transition frequencies are given by the arithmetic mean of the frequencies of the two “interacting” transitions.

Once the simulations had been refined by including the observed crossovers, a custom Python3 routine was used to accurately model the spectral line profiles and to retrieve the experimental transition frequencies. This code is an adaptation of the tool used in ref (41) to model the astrophysical spectra of the amidogen radical isotopologues. To reproduce the recorded spectra, the *1f* and *3f* profile functions are computed for each hyperfine component (also including crossovers), they are summed up, and the resulting profile is optimized versus the experimental spectra in a least-squares fashion. The *1f* and *3f* profile functions are defined as derivatives of the typical dispersive NICE-OHMS signals^42^
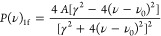

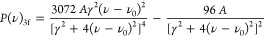
The adjustable parameters are the line position ν_0_, the line intensity *A*, and the width of each component γ. If necessary, a cubic polynomial is also fitted to reproduce the spectral background. The final agreement between the modeled and experimental spectra is fairly good, as shown in Figures 2 and 3. In these plots, the optimized positions and intensities of the hyperfine components and crossovers are depicted as stick spectra.

From the line profile analysis of the spectra recorded, the “best-fit” frequencies were retrieved as explained above; then, they were assigned to the corresponding transitions in terms of quantum numbers and analyzed using the SPFIT routine of the CALPGM suite^43^ and adopting the standard Watson Hamiltonian in the *I*^*r*^ representation.^44^ For blended transitions, the retrieved frequencies were assigned to the intensity-weighted average of the involved components. The analysis of 30 hyperfine frequencies led to the very first determination of the nuclear quadrupole-coupling and spin-rotation constants of ^17^O for vibrationally excited H_2_^17^O. Their values, together with their 3σ uncertainties, are collected in Table 4. From this table, a very good agreement between experiment and theory is noted, with the computed values lying well within the experimental uncertainty. As already pointed out in ref (1), the level of theory employed is able to provide a quantitative prediction of hyperfine parameters. The same accuracy is therefore expected for all computational data reported in Tables 1–3. Since the inclusion of the spin-rotation constants of hydrogen has a negligible effect on the simulation of the rovibrational spectra of *ortho*-H_2_^17^O, such interaction was not considered in the final analysis.

**Table 4:** ^17^O Hyperfine Parameters for H_2_^17^O in the (200) Excited Vibrational Level

parameter^a^	unit	exp.^b^	theory^c^
*χ*_aa_	MHz	–8.86(9)	–8.824
*χ*_bb_	MHz	–1.86(14)	–1.780
*C*_aa_	kHz	–48(16)	–40.89
*C*_bb_	kHz	–38(9)	–33.01
*C*_cc_	kHz	–19(4)	–21.09
no. data		30	
rms	kHz	10.8	

a Being the nuclear quadrupole-coupling tensor traceless, only two χ’s are given.

b Values in parentheses denote 3σ uncertainties in the unit of the last quoted digit.

c Results retrieved from Tables 1 and 2.

As an additional finding, the absolute frequencies of the unperturbed rovibrational transitions recorded in the present study can be retrieved from the analysis. They can be obtained by subtracting the energy contribution due to the hyperfine interactions from the measured hyperfine components. The results of this analysis are presented in Table 5. The absolute accuracy of these transitions is improved by about 2 orders of magnitude with respect to the line frequencies listed in the W2020 database^8^ for H_2_^17^O.

**Table 5:** Absolute Transitions Frequencies of Rovibrational Transitions in the (200) ← (000) Overtone Band of H_2_^17^O

line	frequency (MHz)
1_11_ ← 0_00_	216 698 971.729(20)
0_00_ ← 1_11_	214 541 043.881(22)
2_02_ ← 1_11_	216 570 396.811(10)
2_02_ ← 3_13_	213 423 435.893(10)
6_16_ ← 5_05_	218 793 162.770(34)
8_27_ ← 8_18_	218 532 715.457(17)

## Conclusions

The present study reports on the first observation of hyperfine-resolved rovibrational transitions of the (200) ← (000) overtone band of H_2_^17^O, recorded in the saturation regime by means of the NICE-OHMS technique. Six transitions, four pertaining to the para-species and two to the ortho-species, were investigated and the nuclear quadrupole-coupling and spin-rotation constants of oxygen derived for the (200) vibrational level. A very good agreement, within 3σ, is found with the corresponding computations. As pointed out in refs (1, 2) and confirmed in the present study, quantitative predictions of the hyperfine parameters can be provided, which, however, cannot be obtained routinely. For the first time, the variational DVR-QAK scheme has been extended to compute the vibrational corrections for excited states. Finally, the unperturbed frequencies of the observed rovibrational transitions of H_2_^17^O were determined at an unprecedented accuracy of 20 kHz, corresponding to a relative uncertainty of 10^–11^. These figures supersede by far the quality of the data available in the W2020 database for H_2_^17^O.

## Footnotes

a This is obtained by a least-squares procedure involving experimental ground-state rotational constants of various isotopologues computationally corrected for vibrational contributions (from anharmonic force-field calculations).
